# Attribute Conditioning is insensitive to cue competition and is not predicted by the Big Five Personality Traits

**DOI:** 10.1177/01461672241308921

**Published:** 2025-01-15

**Authors:** Martyn Quigley, Simon Dymond, Katie Kiely, Alex Bradley, Mark Haselgrove

**Affiliations:** 1 Swansea University, UK; 2 Reykjavík University, Iceland; 3 University of Portsmouth, UK; 4 University of Nottingham, UK

**Keywords:** Attribute Conditioning, personality, cue competition, healthiness

## Abstract

When a neutral stimulus is paired with a stimulus denoting an attribute, the neutral stimulus inherits that attribute (i.e., Attribute Conditioning; AC). The current experiments examined whether this effect is sensitive to cue competition, specifically blocking (Experiment 1, *n* = 245) and overshadowing (Experiment 2, *n* = 213), and whether personality traits can predict this effect (*n* = 458). Participants were shown cartoon images of people (CSs) paired with healthy or unhealthy foods (USs) and completed the Big Five Inventory. An AC effect was evident—people paired with healthy foods were rated healthier than people paired with unhealthy foods. However, there was no evidence of cue competition or personality traits impacting the AC effect, although females displayed a stronger AC effect than males. These findings indicate that AC is a robust phenomenon of relevance to social learning processes but is insensitive to factors that influence other forms of conditioning.

When a neutral stimulus is paired with a stimulus denoting a specific attribute, the neutral stimulus can acquire the attribute of the stimulus it is paired with (Unkelbach & Högden, 2019). For example, pairing an image of a neutral food product (e.g., a cereal bar) with an image of someone exercising results in the food being perceived as healthy, whereas pairing a neutral food product with an image of someone drinking alcohol results in the food being perceived as unhealthy (Förderer & Unkelbach, 2015). This effect, termed *Attribute Conditioning* (AC), has been demonstrated using a variety of experimental procedures and stimuli, including faces, words, and foods (Förderer & Unkelbach, 2011, 2014, 2015). AC is separable from other similar forms of conditioning such as Evaluative Conditioning (EC) despite sharing common procedural features. In EC, the valence or likability of a neutral stimulus is changed through repeated pairings with a stimulus that is perceived to have a positive or negative valence (De Houwer et al., 2001; Hofmann et al., 2010). In AC, the neutral stimulus is imbued with a specific attribute or characteristic (e.g., humor, healthiness), yet the valence of the stimulus may remain unchanged (see Förderer & Unkelbach, 2014).

Despite AC and EC effects being orthogonal to one another, the same theoretical accounts have been provided to explain these effects, including the signal learning account (for reviews see Baeyens et al., 1992; De Houwer et al., 2001; Unkelbach & Högden, 2019). According to the signal learning account, AC and EC effects are the products of Pavlovian conditioning with the initially neutral stimulus serving as a conditioned stimulus (CS) and the stimulus denoting a specific attribute serving as an unconditioned stimulus (US). When a CS reliably signals the presence of the US, the CS produces responses that are typically elicited by the US as the CS is assumed to prompt an expectation of the US’ impending presentation (Pearce & Bouton, 2001). On this basis, then, the properties of standard Pavlovian conditioning should be reflected in AC. For example, varying the statistical contingency between the CS and US should therefore influence the presence of AC or EC effects in the same way that it does Pavlovian conditioning with both human and non-human animals (e.g., Rescorla, 1968). Evidence in support of this account is heterogeneous though (see De Houwer et al., 2001; additional accounts are considered in the discussion). While some conditioning effects consistent with the signal learning account (e.g., counterconditioning and CS and US pre-exposure effects) have been reported in EC procedures (Baeyens et al., 1989; Hammerl et al., 1997; Stuart et al., 1987), cue competition effects such as blocking have not been observed in either EC or AC procedures (Dwyer et al., 2007; Förderer & Unkelbach, 2015).

The blocking effect (Kamin, 1968, 1969; Le Pelley et al., 2005) has been critical to the development of key models of learning and conditioning (e.g., Rescorla & Wagner, 1972) as it reveals how the associative history of CSs interact to influence learning. In a blocking study, a CS is reliably paired with a US (A+) in a first stage of training before being presented in compound with a second CS and predicting the same US (i.e., AB+) in a second stage of training. Blocking occurs when learning about the relationship between the second CS (B) and the US is “blocked” (i.e., attenuated) due to prior conditioning between the first CS (A) and the US. To determine whether blocking has occurred, learning about the blocked cue (B) is typically compared with learning about an element (D) of a control compound CS (CD+) which is paired with a US, but neither element of the compound has been previously predictive of the US (i.e., Kamin control trials; Kamin, 1968, 1969). For example, in the context of an AC task, we might arrange for participants to see trials in which a picture of a neutral person, Alex, is paired with a US that denotes the attribute of healthiness. In a second stage of the experiment, Alex would be presented together with a new person, Blake, and again paired with healthiness. These trials would be intermixed, in Stage 2, with trials in which Charlie and Dani are also paired with healthiness. Blocking would be observed if, following this training, people judged Dani to have a greater attribute of healthiness than Blake, even though these two people were paired with healthiness equally often. This result follows from the signal learning analysis of AC as the prior learning about Alex establishes Blake as a redundant signal for healthiness, limiting its associability.

In two experiments, Förderer and Unkelbach (2015) investigated whether blocking could be observed in an AC procedure with the attributes of health and athleticism and found no evidence of blocking. Blocking was indexed by examining differences in participants’ attribute ratings between the blocking (e.g., A) and blocked stimuli (e.g., B); however, the control trials that are typically incorporated into blocking studies were not included (e.g., Kamin control trials). As such, it is unclear whether blocking can be obtained in an AC task when traditional control procedures are employed to index the effect. Furthermore, relatively small sample sizes were used in these previous experiments (*n* < 50) which can lead to both false positives and false negatives (Button et al., 2013; Francis, 2012). Interestingly, alternative cue competition effects such as overshadowing (Pavlov, 1927) also appear not to have been tested in an AC task. Overshadowing, like blocking, is deemed to arise because of a competitive learning principle. The key distinction between these effects lies in the procedural arrangement of the stimuli. An overshadowing effect occurs when the relationship between a stimulus presented alone and a US (e.g., A+) is learned about better than the relationship between one or both elements of a compound stimulus (e.g., BC+) and the same US (i.e., Mackintosh, 1976; Prados, 2011; Quigley et al., 2023).

Considering the above, Experiment 1 sought to examine whether blocking could be obtained in an AC task using typically employed control procedures and appropriately powered sample sizes; whereas Experiment 2 sought to examine whether overshadowing could be observed. If blocking and overshadowing are observed under these conditions, this would provide evidence in support of the signal learning account of AC. In addition, we also assessed whether conditioning a specific attribute (i.e., health) influenced participants’ ratings of other nonconditioned attributes (i.e., athleticism, education, humor, and organization). That is, whether there was an indirect effect of conditioning the attribute of health on participants’ ratings for other attributes and what the nature of any indirect effect may be. It is possible, for instance, that a “halo” effect is observed where participants rate the CSs paired with healthy foods more positively for all the nonconditioned attributes that have positive connotations (Nisbett & Wilson, 1977; see Unkelbach & Högden, 2019).

Finally, we also sought to assess whether the AC effect could be predicted by the personality traits specified in the Big Five model of personality: Openness, Conscientiousness, Extraversion, Agreeableness, and Neuroticism (see Digman, 1990; Goldberg, 1993). Openness reflects a person’s creativity and curiosity. Conscientiousness represents a tendency to be organized and responsible. Extraversion describes how outgoing and social a person is. Agreeableness involves being trusting and cooperative, whereas neuroticism refers to a tendency to experience anxiety and stress. Recent research has begun to analyze the relationship between these personality traits and EC, which is conceptually similar to AC. For example, both Vogel et al. (2019) and Ingendahl and Vogel (2023) found that higher levels of agreeableness and neuroticism resulted in stronger EC. Interestingly, Ingendahl and Vogel (2023) reported that participants scoring higher in agreeableness displayed more extreme evaluations of USs and had better memory about the stimuli pairings, which may account for the enhanced EC effect. These authors also note, however, that agreeable participants may simply be more amenable to conditioning. Regarding neuroticism, the authors postulated that higher levels of this trait may result in an enhanced focus on the valence of stimuli, thus strengthening EC. Together these findings suggest that (at least some of) the Big Five personality traits influence EC. To our knowledge, however, there is no research that has currently examined the relationship between the Big Five personality traits and AC. As such, we sought to also examine this as part of our study.

## Experiment 1: Blocking

To assess whether blocking could be observed in AC, a task was administered to condition the attribute of healthiness that was based on the final experiment of Förderer and Unkelbach (2015). First, participants provided ratings of healthy or unhealthy foods (which served as USs) during a *preratings* stage. Then, neutral images of cartoon people (i.e., the CSs) were paired with foods that were either explicitly healthy or unhealthy. During a first stage of training, individual cartoon images (i.e., elemental CSs; i.e., A, B) were paired individually with healthy (US1) or unhealthy (US2) foods, before these CSs were presented in compound with additional CSs during a second stage of training (i.e., AW, BX). In the second stage of training, compound control trials (i.e., CY and DZ) were also presented and paired with healthy or unhealthy foods in an identical fashion to AW and BX, respectively (see Table 1). Participants were then asked to rate the healthiness of each of the CSs (individually) during a test stage and, in addition, to provide ratings of how athletic, educated, organized, and humorous they perceived each CS to be. This was done to assess whether an AC effect was evident and to examine whether conditioning the attribute of health influenced participants’ ratings of other nonconditioned attributes. If an AC effect is evident, it would be expected that participants would provide higher health ratings to the cartoon images paired with the healthy foods than those paired with the unhealthy foods. If conditioning the attribute of health had an impact on nonconditioned attributes, differences would also be expected between nonconditioned attribute ratings for the CSs paired with healthy foods and those paired with unhealthy foods. If a blocking effect is observed, higher ratings would be expected for the control CSs paired with healthy foods than the blocked CSs paired with healthy foods. Equally, control CSs paired with unhealthy foods would be rated unhealthier than blocked CSs paired with unhealthy foods. We predicted that an AC effect would be evident based on previous studies. However, given limited previous research, our analyses were exploratory in relation to the possible presence of blocking in an AC task and the impact of conditioning the attribute of health on attributes that had not been directly conditioned.

**Table 1.** Design of Experiments 1 and 2.

Experiment	*N*	Trial type	Preratings	Training (Stage 1)	Training (Stage 2)	Test ratings
Exp. 1 (Blocking)	*245*	Blocking	USs	A—US1: Healthy	AW—US1: Healthy	A–Z: Athletic
				B—US2: Unhealthy	BX—US2: Unhealthy	A–Z: Educated
						A–Z: **Health**
		Control		-	CY—US1: Healthy	A–Z: Humorous
				-	DZ—US2: Unhealthy	A–Z: Organized
Exp. 2 (Overshadowing)	*213*	Control	CSs & USs	A—US1: Healthy	-	A–Z: Athletic
				B—US2: Unhealthy	-	A–Z: Educated
						A–Z: **Health**
		Overshadowing		CY—US1: Healthy	-	A–Z: Humorous
				DZ—US2: Unhealthy	-	A–Z: Organized

*Note. N* = sample size. A–Z refer to the cartoon images (i.e., CSs), while US1 and US2 refer to the healthy and unhealthy foods. Athletic, Educated, Health, Humor, and Organized denote the attributes participants provided ratings for at the test stage.

## Method

The methodology and materials file and datasets for all experiments reported in this manuscript are publicly available at the Open Science Foundation: https://osf.io/xmju7/?view_only=3e61072eb0ea4a718f76d5b8876c4f39. These experiments were not preregistered.

### Participants

A total of 245 participants were recruited from Swansea University’s School of Psychology and the local community: Age (*M* = 21.35; *SD*: 7.29; *range*: 18 – 65 years old); sex: *female* = 181 (73.88%): *male =* 64 (26.12%). Participants received participant-pool credits for their participation or took part voluntarily. Ethical approval was provided by Swansea University’s Ethics Committee. The sample size was based on an a priori power calculation using G*Power 3.1 (Faul et al., 2009). To detect a small effect (Cohen’s *f* = .10) of AC and blocking with two within-subjects measurement (taken during the test stage), α =.05 and Power (1 – β) =. 80, results indicated a total of 199 participants would be needed.

### Stimuli and Materials

We used Gorilla Experiment Builder (Anwyl-Irvine et al., 2018) to administer the task and questionnaires online. Participants could use a desktop computer, a laptop, or a tablet/mobile phone to access the study.

**Figure 1.**
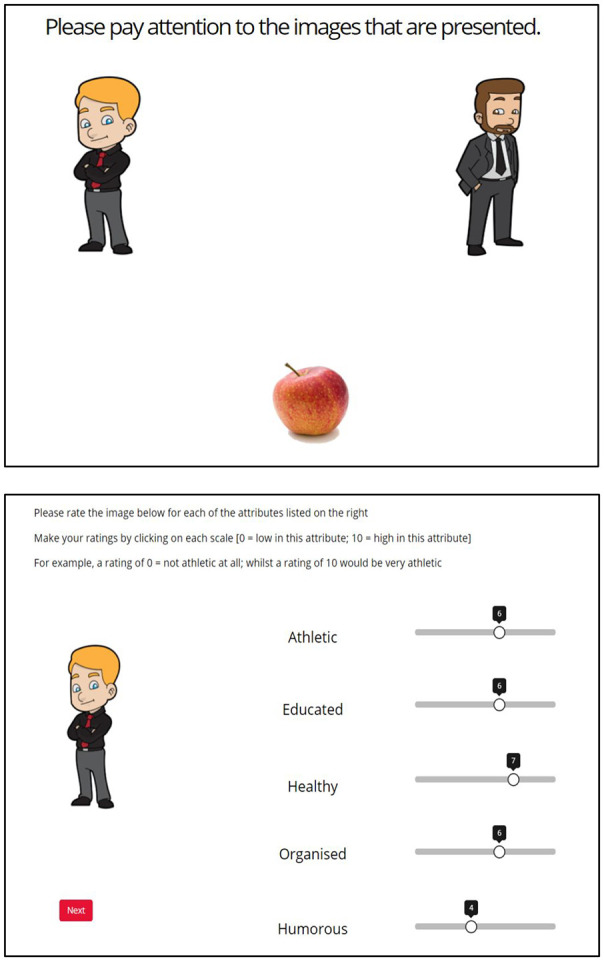
Example of a Trial From the Training Stage Featuring Two CSs (Top) and an Example Trial From the Test Stage (Bottom).

Images of cartoon people and real foods (see Figure 1) were assigned to the CSs (i.e., letters A–Z) and USs (i.e., US1 and US2) in Table 1 using a Latin-Square counterbalancing technique. The cartoon images always served as the CSs A–Z, and the healthy and unhealthy food always served as US1 and US2, respectively. There were eight cartoon images of people which served as CSs A–Z. The cartoon images of people were all wearing professional attire and were balanced for sex (see Figure 1, for example). All images were the same size. The healthy US was a picture of an apple, and the unhealthy US was a picture of a donut.

In the *preratings stage*, participants were presented with the USs individually in the center of the screen. Participants were asked to rate how healthy each stimulus was by moving a cursor on a Likert-type scale which ranged from 0 to 10 [“0” = “Unhealthy,” “10” = Healthy”] that was positioned toward to the bottom of the screen.

In the *training stage*, participants were presented with either a single cartoon image left or right of center in the top half of the screen (i.e., an elemental CS trial) or two cartoon images in the center of the top half of the screen (i.e., a compound CS trial), against a white background. The position of the cartoon images on the screen (i.e., left or right of center) was counterbalanced across trials. The USs (i.e., an image of the food) were then presented in the center of the bottom half of the screen.

In the *test stage*, participants were asked to rate how athletic, educated, healthy, humorous and organized each of the cartoon images were on a 0 to 10 Likert-type scale [0 = “Low in this attribute,” 10 = “High in this attribute”]. Each of the cartoon images were presented individually toward the left of the screen and the five attributes were positioned on the right of the screen in text form (see Figure 1, for example). Participants made their ratings for each attribute by moving a cursor on each of the 0 to 10 Likert-type scales that were positioned next to each of the attributes.

### Design and Procedure

An online link was provided to participants that took them to an information sheet and consent form, before demographic details were requested (i.e., age and sex). Once demographic details had been provided, participants were then presented with the following instructions before progressing to the preratings stage: “Before the task begins you will be asked to rate how healthy certain foods and cartoon people are on a scale ranging from 0 – 10 [0 = Unhealthy; 10 = Healthy].” Participants were then presented with each of the stimuli in the center of the screen and required to provide a rating on the Likert-type scale before advancing to the next screen. Once the preratings were provided, participants were then presented with the following instructions before progressing to the training stage:

Please make sure to pay attention to the following images that are presented. You do not need to do anything in this stage but to pay attention. The task may be a little repetitive at times, but it won’t take too long (approx. 10 mins). We really do appreciate your help!

Across the training stages of both experiments, 32 trials were presented. The order of trials was randomized.

In Training Stage 1 of Experiment 1, participants were presented with eight A—US1 trials and eight B—US2 trials. In Training Stage 2, participants received four trials of each CS–US pairing (AW–US1, BX–US2, CY–US1, DZ–US2). There was no break between the two training stages.

On each trial, a fixation cross was first presented for 1 s before the CS/s were presented alone (top half of the screen) for 1 s before being accompanied by the US for 2.5 s (bottom half of the screen). Each trial began immediately after the previous trial; no response was required from participants to initiate the trial. When all 32 trials from the training stages were complete, participants proceeded to the test stage.

When commencing the test stage participants were asked to “Please rate the image below for each of the attributes listed on the right. Make your ratings by clicking on each scale [0 = Low in the attribute; 10 = High in this attribute].” Below this text, each of the CSs (i.e., cartoon images) were presented individually, one per screen (see Figure 1). Next to each cartoon image (to the right), five Likert-type rating scales were presented, one for each attribute: *Athletic*, *Educated*, *Healthy*, *Humorous*, and *Organized*. Participants were required to make a rating for each attribute. Once they had rated all the CSs for each attribute, participants were then presented with the following text “what did you think the purpose of this task was? [if you are unsure simply type ‘unsure’]” they were then provided with a free text response box where they could type their response. Once participants provided their response, they were then presented with text informing them that they would be asked to complete a short questionnaire. The Big Five Inventory (BFI; John & Srivastava, 1999) then followed (see additional analyses section for further details), before participants were presented with a debrief form.

### Data Analysis

All datasets can be found on the Open Science Framework: https://osf.io/xmju7/?view_only=3e61072eb0ea4a718f76d5b8876c4f39. All analyses were conducted using JASP version 18.3 (JASP Team, 2024). Data were analyzed using both frequentist and Bayesian paired-samples *t*-tests, mixed model analyses of variances (ANOVAs) and multiple linear regressions. For all frequentist analyses, an alpha (α) of .05 was adopted unless otherwise stated. For all frequentist ANOVAs, Greenhouse–Geisser corrected *F*-ratios and degrees of freedom are reported (when the assumption of sphericity was not met). Bayesian analyses were undertaken using default priors to estimate the Bayes Factor_10_ (BF_10_; Rouder et al., 2012). As such, the weight of evidence for the alternative hypothesis over the null (BF_10_) was examined where values >1, <1, and = to 1, respectively, represent increasing evidence for the alternative hypothesis, increasing evidence for the null hypothesis, and evidence for neither hypothesis (Lee & Wagenmakers, 2013).

## Results

### Preratings Stage

A paired-samples *t*-test revealed, as expected, that the healthy US (*M* = 9.11; *SD* = 1.16) was rated healthier than the unhealthy US (*M* = 1.29; *SD* = 1.64), *t* (244) = 59.22, *p* < .001, Cohen’s *d* = 3.78, 95% confidence interval (CI) = [3.42, 4.14], BF_10_ > 100.

### Test Stage: AC

**Figure 2.**
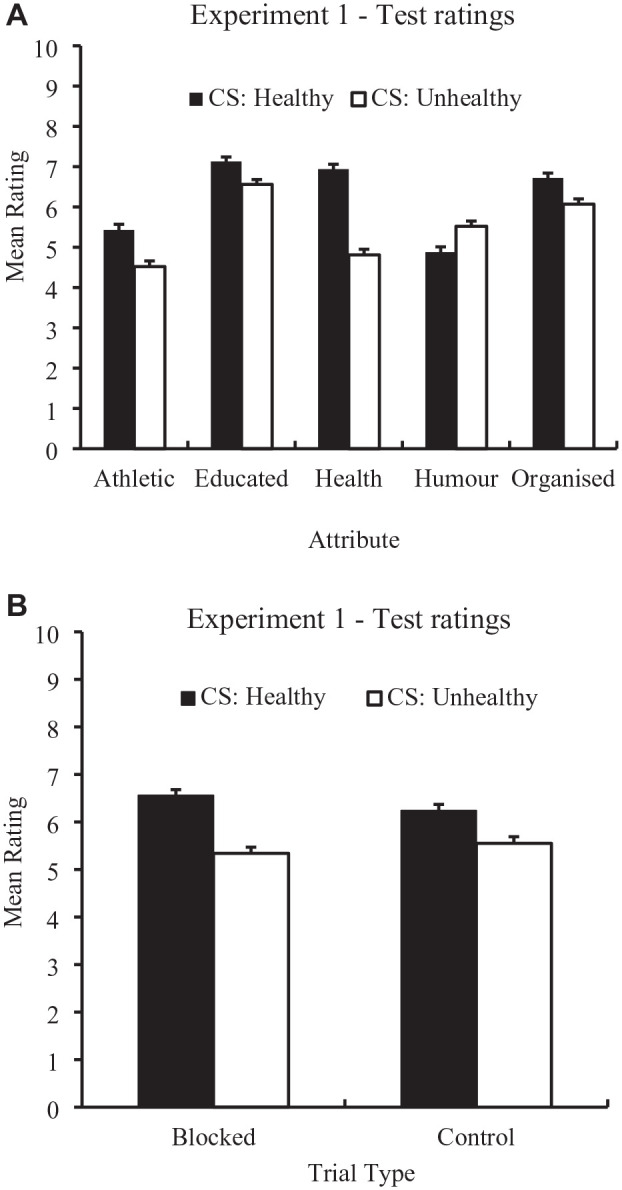
Mean Ratings for the Healthy and Unhealthy CSs for All Attributes (Top) and Mean Health Ratings for the “Blocked” (W and X) and Control (Y and Z) CSs (Bottom). Error bars represent SEM.

To examine whether an AC effect was present, participants’ attribute ratings at the test stage to Stimulus A (which was always paired with the healthy US) and Stimulus B (which was always paired with the unhealthy US) were analyzed. Figure 2 (top panel) displays the means (and SEM) of the test ratings for these stimuli (i.e., Stimulus A = “CS: Healthy”; Stimulus B = “CS: Unhealthy”) for each attribute. To analyze these data, a 2 × 5 within-subjects ANOVA of individual ratings was conducted with the factors of Stimulus (CS: Healthy vs. CS: Unhealthy) and Attribute (Athletic, Educated, Health, Humor, Organized). There was a main effect of Stimulus, *F*(1, 244) = 41.48, *p* < .001, η_p_^2^ = .15, 95% CI [.07, .23], BF_10_ > 100, and Attribute, *F*(3.26, 794.12) = 99.26, *p* < .001, η_p_^2^ = .29, 95% CI [.25, .36], BF_10_ > 100 and a Stimulus × Attribute interaction, *F*(3.11, 759.16) = 42.55, *p* < .001, η_p_^2^ = .15, 95% CI [.11, .19], BF_10_ > 100. Simple main effects revealed that “CS: Healthy” received higher *health* ratings than “CS: Unhealthy,” *F*(1, 244) = 113.65, *p* < .001, *d* = .68. Higher ratings were also provided to “CS: Healthy” for the attributes: athleticism, *F*(1, 244) = 21.90, *p* < .001, *d* = .30, education, *F*(1, 244) = 20.80, *p* < .001, *d* = .29, and organization, *F*(1, 244) = 15.53, *p* < .001, *d* = .25; however, ratings were lower for the attribute humor, *F*(1, 244) = 12.06, *p* < .001, *d* = .22.

To determine attribute specificity (i.e., that there was a specific effect of the attribute of health being conditioned) Bonferroni corrected paired-samples *t*-tests were conducted comparing the difference in *health* ratings between “CS: Healthy” and “CS: Unhealthy” versus the difference in ratings for “CS: Healthy” and “CS: Unhealthy” for each of the four nonconditioned attributes (i.e., Förderer & Unkelbach, 2014). The difference in *health* ratings between “CS: Healthy” and “CS: Unhealthy” was greater than the difference in ratings of athleticism, *t*(244) = 7.17, *p* < .001, *d* = .46, BF_10_ > 100; education, *t*(244) = 7.93, *p* < .001, *d* = .51, BF_10_ > 100, humor, *t*(244) = 5.25, *p* < .001, *d* = .34, BF_10_ > 100; and organization, *t*(244) = 7.40, *p* < .001, *d* = .47, BF_10_ > 100, thus demonstrating a specific AC effect of the attribute health.

### Test Stage: Blocking

To examine whether a *blocking* effect was present for the healthy CS, participants’ health ratings at the test stage to Stimulus W (the “blocked” CS paired with the healthy US) was compared with Stimulus Y (the control CS which was paired with the healthy US). To examine whether a blocking effect was present for the unhealthy CSs, participants’ health ratings at the test stage to Stimulus X (the “blocked” CS paired with the unhealthy US) was compared with Stimulus *Z* (the control CS which was paired with the unhealthy US). Mean ratings (and SEM) for these stimuli are presented in Figure 2. A 2 (Attribute: healthy vs. unhealthy) × 2 (Stimulus: blocked vs. control) within-subjects ANOVA of individual ratings revealed no main effect of stimulus, *F*(1, 244) = .25, *p* = .62, η_p_^2^ = .00, 95% CI = [.00, .02], BF_10_ = .09, but a main effect of Attribute, *F*(1, 244) = 42.59, *p* < .001, η_p_^2^ = .15, 95% CI = [.07, .24], BF_10_ > 100, with the healthy stimuli receiving healthier ratings than the unhealthy CSs, and a Stimulus × Attribute interaction, *F*(1, 244) = 5.84, *p* < .05, η_p_^2^ = .02, 95% CI = [.00, .07], BF_10_ = 2.63. Simple main effects revealed that ratings for the “blocked” and control stimuli did not differ in the unhealthy condition, *F*(1, 244) = 1.78, *p* = .18, *d* = .09, BF_10_ = .17.^1^ However, the “blocked” stimulus was rated higher than the control stimulus in the healthy condition, *F*(1, 244) = 4.44, *p* <.05, *d* = .14, BF_10_ = .63.^2^

## Discussion

Participants received trials in which neutral stimuli (cartoon representations of different people) were paired with a healthy or unhealthy food. Consistent with the previous literature (Förderer & Unkelbach, 2015), Experiment 1 demonstrated a robust AC effect in which ratings of the health of the person aligned with the healthiness of the food with which they had been paired. Conditioning the attribute of health also influenced nonconditioned attribute ratings: the CS paired with the healthy food was rated more athletic, educated, and organized—but less humorous—than the CS paired with the unhealthy food (for further discussion see the General Discussion). However, there was no evidence of blocking; in fact, the results indicated a small but significant augmentation effect (Batsell & Baston, 1999) where the “blocked” stimulus received a higher rating than the control stimulus in the healthy condition. The current experiment was not designed to understand *why* an augmentation, rather than a blocking effect was observed. However, it is notable that Vadillo and Matute (2010) have demonstrated augmentation in participants when the cue duration was short (3 s), whereas blocking was demonstrated when the cue duration was long (6 s); and in the current experiment, cue duration was relatively short (1 s). In addition, we can look toward studies of augmentation of learning in rats, which suggest that the effect is a consequence of the formation of within-compound associations. Allswede et al. (2014), for example, showed that giving A-US1 trials in Stage 1 prior to AW-US1 trials in Stage 2 significantly augmented learning about W in a flavor aversion procedure. They proposed that the augmented response to W was a consequence of the formation of a within-compound association between W and A during Stage 2. The outcome of this association would be that upon testing W, the conditioned response would not solely be a function of the associative strength of W, but also any associative strength that A has acquired, and which was accessed by W through the W-A-O1 associative chain. In support of this analysis, Allswede et al. demonstrated that presenting A in the absence of O1 (i.e., extinguishing it) after Stage 2 significantly attenuated augmentation, showing that the effect was (at least partly) a consequence of the associative status of A. Perhaps, then, the conditions of training in Stage 2 of Experiment 1 were amenable for the acquisition of a within-compound association between W and A, and so at test presentation of W activated a representation of US1 through the spread of activation along the W-A-US1 associative chain.

As appealing as the foregoing explanation is for the presence of augmentation, a limitation of the analysis is that, in its simplest form, it does not provide a ready explanation for why augmentation was observed only to stimuli that were paired with the healthy US, but not the unhealthy US. Additional assumptions must therefore be made. In any case, the failure to observe blocking begs the question as to whether other, related, cue competition effects are attenuated in AC. The purpose of Experiment 2 was to explore this.

## Experiment 2

Experiment 2 sought to further explore the relationship between AC and cue competition effects to determine whether the effect observed in Experiment 1 is specific to blocking, or whether they extend to other cue competition phenomena. In Experiment 2, we examined the cue competition effect of overshadowing (Pavlov, 1927) which has provided important insights into the development of conditioning models (e.g., Mackintosh, 1976). Here, two CSs are presented in compound (i.e., together) and paired with an outcome (AB+). Overshadowing is said to occur when the relationship between these CSs and the outcome is learned about less well than a control CS which is simply paired with the outcome on its own (C+; e.g., Mackintosh, 1976; Prados, 2011; Quigley et al., 2023). To our knowledge, overshadowing has not been explored in an AC task. If an overshadowing effect is observed participants would learn about the relationship between CSs and a US better if the CS is conditioned in isolation than if it is conditioned in compound with a second CS. In addition, in this experiment, participants provided preratings of the health of CSs and USs rather than just USs (as in Experiment 1). This change was introduced to ensure that participants did not have preconceived ideas concerning the healthiness of the CSs that would be paired with the USs which would therefore bias their ratings.

## Method

### Participants

A total of 213 participants took part: Age (*M* = 20.49; *SD*: 4.56; *range*: 18–53 years old); sex: *female* = 158 (74.18%); *male =* 50 (23.47%); nonbinary = 5 (2.35%). All other details were the same as Experiment 1.

### Stimuli and Materials

The stimuli and materials were identical to Experiment 1, except that only six CSs (i.e., cartoon images) were used as opposed to the eight in Experiment 1.

### Design and Procedures

The design of Experiment 2 can be seen in Table 1. All details were the same as Experiment 1, with the exception that in this experiment CSs were presented in the preratings stage in addition to USs. Also, a single training stage was presented where participants were presented with eight trials of each CS–US pairing (A—US1, B—US2, CY—US1, DZ—US2).

## Results

### Preratings

Paired-samples *t*-tests revealed that ratings of the CSs that would be paired with the healthy US (*M =* 7.11; *SD* = 1.59) and the unhealthy US (*M =* 7.13; *SD* = 1.53) were comparable, *t*(212) = −.42, *p* = .68, Cohen’s *d* = −.03, 95% CI = [−.16, .11], BF_10_ = .08.

However, the healthy US (*M* = 9.08; *SD* = 1.29) and unhealthy US (*M =* 1.20; *SD* = 1.43) were rated differently as expected, *t*(212) = 55.05, *p* < .001, Cohen’s *d* = 3.77, 95% CI = [3.38, 4.15], BF_10_ > 100.

### Test Stage: AC

**Figure 3.**
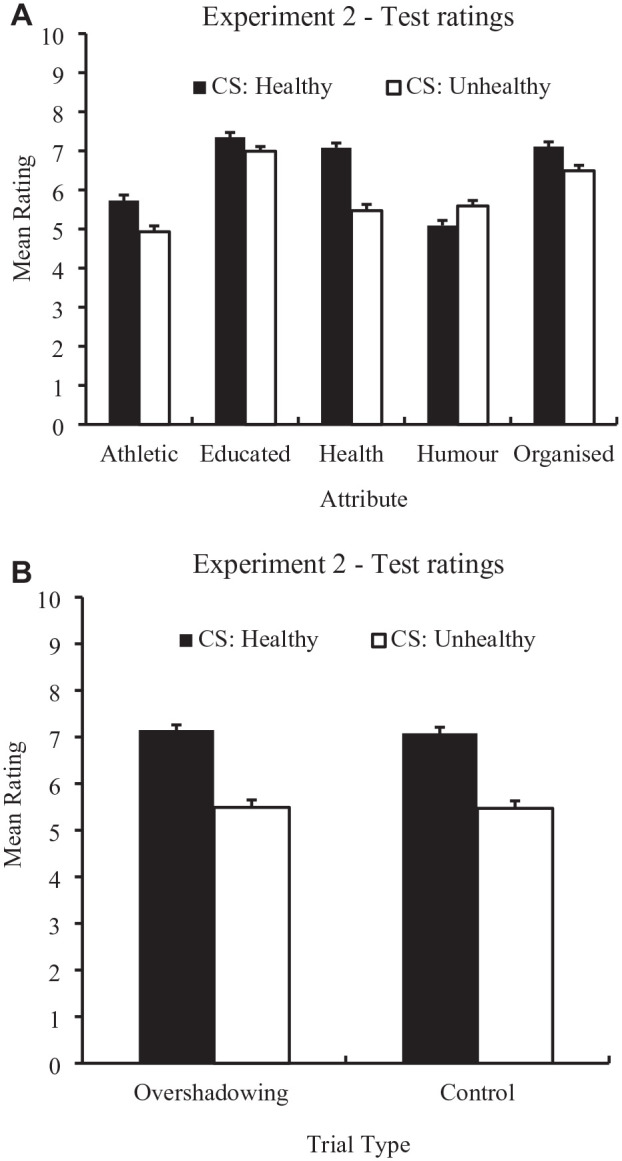
Mean Ratings for the Healthy and Unhealthy CSs for All Attributes (Top) and Mean Health Ratings for the Overshadowing (Y and Z) and Control (A and B) CSs (Bottom). Error bars represent SEM.

Figure 3 (top panel) displays the means (and SEM) of the test ratings for Stimuli A and B (i.e., Stimulus A = “CS: Healthy”; Stimulus B = “CS: Unhealthy”) for each attribute. An identical ANOVA to that conducted for the test stage of Experiment 1 was conducted for Experiment 2. There was a main effect of Stimulus, *F*(1, 212) = 24.23, *p* < .001, η_p_^2^ = .10, 95% CI [.04, .19], BF_10_ > 100 and Attribute, *F*(2.99, 633.94) = 102.92, *p* < .001, η_p_^2^ = .33, 95% CI [.26, .38], BF_10_ > 100 and a Stimulus × Attribute interaction, *F*(3.22, 682.99) = 25.72, *p* < .001, η_p_^2^ = .11, 95% CI [.07, .15], BF_10_ > 100. Simple main effects revealed that Stimulus A (i.e., “CS: Healthy”) received higher health ratings than Stimulus B (i.e., “CS: Unhealthy”), *F*(1, 212) = 57.15, *p* < .001, *d* = .52. Higher ratings were also provided for “CS: Healthy” compared with “CS: Unhealthy” for the attributes: athleticism, *F*(1, 212) = 16.88, *p* < .001, *d* = .28; education, *F*(1, 212) = 7.08, *p* < .01, *d* = .18; and organization, *F*(1, 212) = 13.14, *p* < .001, *d* = .25. However, ratings were lower for the attribute humor, *F*(1, 212) = 8.91, *p* < .01, *d* = .21. Bonferroni-corrected paired-samples *t*-tests revealed that the difference in health ratings between “CS: Healthy” and “CS: Unhealthy” was greater than the difference in ratings of athleticism, *t*(212) = 4.23, *p* < .001, *d* = .29, BF_10_ > 100; education, *t*(212) = 6.13, *p* < .001, *d* = .42, BF_10_ > 100; humor, *t*(212) = 4.10, *p* < .001, *d* = .28, BF_10_ > 100; and organization, *t*(212) = 4.69, *p* < .001, *d* = .32, BF_10_ > 100, thus demonstrating a pattern of results consistent with Experiment 1.

### Test Stage: Overshadowing

To examine whether an *overshadowing* effect was present between the healthy CSs, participants’ health ratings to Stimulus Y (the “Overshadowed” CS paired with the healthy US) was compared with Stimulus A (the control CS which was paired with the healthy US). To examine whether an overshadowing effect was present for the unhealthy CSs, participants’ health ratings at the test stage to Stimulus *Z* (the “Overshadowed” CS paired with the unhealthy US) was compared with Stimulus B (the control CS which was paired with the unhealthy US). Mean ratings for these stimuli are presented in Figure 3. A 2 (Attribute: healthy vs. unhealthy) × 2 (Stimulus: Overshadowed vs. Control) within-subjects ANOVA revealed no main effect of stimulus, *F*(1, 212) = .25, *p* = .62, η_p_^2^ = .00, 95% CI = [.00, .03], BF_10_ = .09, or a Stimulus × Attribute interaction, *F*(1, 212) = .06, *p* = .82, η_p_^2^ = .00, 95% CI = [.00, .02], BF_10_ = .11. However, there was a main effect of Attribute, *F*(1, 212) = 84.26, *p* < .001, η_p_^2^ = .28, 95% CI = [.18, .39], BF_10_ > 100, with the healthy CSs being rated healthier than the unhealthy CSs.

## Discussion

In Experiment 2, participants received trials in which neutral CSs were paired with a healthy or unhealthy US. Consistent with the previous literature (Förderer & Unkelbach, 2015) and Experiment 1, a robust AC effect was observed—health ratings of the CSs aligned with the healthiness of the food with which they were paired. Participants’ preratings of the CSs also confirmed that the healthy and unhealthy CSs did not inherently differ prior to conditioning. Like Experiment 1, conditioning the attribute of health also influenced nonconditioned attribute ratings with the CS paired with the healthy food also being rated more athletic, educated, and organized—but less humorous—than the CS paired with the unhealthy food. There was, however, no evidence of overshadowing—the magnitude of AC was equivalent in CSs conditioned in isolation to those conditioned in compound with another CS, and this result was evident for CSs that were paired with either healthy or unhealthy USs. These results, then, are conceptually consistent with the results of Experiment 1, which also failed to observe a cue competition effect. It is, however, worth noting a slight difference between the results of the current two experiments. In Experiment 1, an augmentation effect was observed to the “blocked” CS that was paired with the healthy US. That is to say, instead of a cue competition effect being observed with this design, the opposite was observed, a cue facilitation effect. We could detect no evidence for a comparable cue facilitation effect in Experiment 2, which would have been evidenced as higher ratings for CS conditioned in compound with the healthy US than the CS conditioned alone. This is not to say that overshadowing designs have failed to observe cue facilitation more generally; studies of conditioning in animals (e.g., Batsell et al., 2012; Durlach & Rescorla, 1980) and action-outcome learning in humans (e.g., Alcalá et al., 2023) have revealed that compound training can facilitate conditioning to the CSs within a compound relative to a CS conditioned in isolation. Like augmentation, potentiation is affected by temporal variables (for a review see Urcelay, 2017). Furthermore, like augmentation, potentiation has also been accounted for in terms of within-compound associations (Durlach & Rescorla, 1980). It remains to be determined, then, why we obtained augmentation of AC in Experiment 1, but no evidence for potentiation of AC in Experiment 2 when a healthy food was employed as the US. It is possible that, for some reason, the spread of activation from the test CS to its associate was more successful in Experiment 1 than in Experiment 2. For example, in Experiment 1, CS A was paired with the US alone during Stage 1 before being conditioned in compound with CS W during Stage 2, and for this reason might be expected to have a significant amount of associative strength. In Experiment 2, however, “overshadowing” CSs were never paired with the US alone. Perhaps, this difference between the experimental designs led to variations in the magnitudes of augmentation and potentiation.

## Additional Analyses: Personality Traits

Here, we assessed the relationship between the Big Five personality traits and the AC effect. To our knowledge, previous research has not examined this relationship. However, recently, studies have begun to examine the relationship between the Big Five personality traits and EC. For instance, Vogel et al. (2019) found that scoring higher in agreeableness and neuroticism resulted in greater EC effects. However, extroversion, openness, and conscientiousness were not related to the EC effect (also see Ingendahl & Vogel, 2023). Given the absence of research on the relationship between personality traits and AC, we examined this by combining the samples from the previous two experiments (recall that the BFI was administered as part of the previous two experiments). Combining the samples from the previous two experiments allowed us to produce an overall sample size that would be more sensitive to detect any relationships between the Big Five personality traits and AC (this approach was also adopted in Vogel et al. (2019)). In addition, we also examined the impact of other individual differences on the AC effect such as age, sex, and participants’ awareness of the purpose of the task, that is to say, the possible influence of demand characteristics on AC (see Unkelbach & Högden, 2019, for further discussion).

## Method

### Participants

The samples from Experiment 1 and Experiment 2 were combined resulting in a total of 458 participants. Age (*M* = 20.95; *SD*: 7.29; *range*: 18–65 years old); sex: *Female* = 339 (74.02%); *Male =* 114 (24.89%); *Non-Binary* = 5 (1.09%). To detect a small effect size of the Big Five personality traits, age, sex, and demand characteristics on AC (Cohen’s *f*^2^ = .02), with an alpha (α) of .05 and power (1 – β) of .80, G*Power 3.1 indicated that 395 participants would be needed using a multiple regression with eight predictors and a single coefficient. All other details were the same as the previous studies.

### Stimuli and Materials

The BFI (John & Srivastava, 1999) was administered in each of the previous experiments. The BFI contains 44 items that measure the following five personality traits: Conscientiousness, Agreeableness, Neuroticism, Openness, and Extraversion. Each item contains a statement (e.g., “I am someone who is talkative”) that participants provide responses to on a 5-point Likert-type scale (1 = *disagree strongly*; 5 = *agree strongly*). The scale has good psychometric properties (John & Srivastava, 1999).

### Design and Procedures

See details of Experiments 1 and 2. Recall that participants first completed the conditioning stages of the task before being asked about what they thought the purpose of the task was and completing the BFI (John & Srivastava, 1999). Specifically, participants were presented with the following text to assess the possible influence of demand characteristics on AC “what did you think the purpose of this task was? [if you are unsure simply type ‘unsure’]” where they could provide a free text response. Participants’ responses were dummy-coded (0 = did not understand purpose of task; 1 = understood purpose of task) by two members of the research team to assess whether participants understood the purpose of the task. Cohen’s kappa (κ) was .52, representing moderate agreement. Disagreements were resolved through discussion.

### Results

Table 2 displays mean scores (and standard deviations) for the subscales of the BFI, age and the size of the AC effect collapsed across Experiments 1 and 2 (i.e., the difference between the health ratings for Stimulus A, which was always paired with the healthy US, and Stimulus B, which was always paired with the unhealthy US). Table 2 also displays the Pearson correlation coefficients between these variables, in addition to point biserial correlations with sex (0 = Female; 1 = Male) and demand characteristics (0 = not understood; 1 = understood).

**Table 2.** Pearson Correlations Between the Big Five Personality Traits, Age, and the Size of the Attribute Conditioning Effect Across Both Experiments and Point Biserial Correlations Between These Variables and Sex (0 = Female; 1 = Male) and Demand Characteristics (0 = Not Understood; 1 = Understood).

Scale	*M* (*SD*)	1	2	3	4	5	6	7	Sex	Demand characteristics
*Openness (1)*	31.74 (5.21)	—	−.10*	.18***	.09*	−.08	.11*	−.01	.08	.02
*Conscientiousness (2)*	29.95 (5.90)		—	.07	.31***	−.18***	.22***	−.01	−.06	.13**
*Extraversion (3)*	25.65 (6.36)			—	.12**	−.32***	.07	.00	−.09	.06
*Agreeableness (4)*	33.69 (5.88)				—	−.21***	.03	.07	−.08	.07
*Neuroticism (5)*	26.53 (6.51)					—	−.14**	−.00	−.26***	−.04
*Age (6)*	20.95 (6.18)						—	−.07	.16***	.06
*AC Effect size (7)*	1.89 (3.12)							—	−.11*	−.01

*Note*. * denotes statistical significance <.05; ** denotes statistical significance <.01; *** denotes statistical significance <.001.

To examine whether these variables could predict the magnitude of AC, a standard multiple regression (using the “enter” method) was performed with personality traits (i.e., the Big Five), age, sex (0 = Female; 1 = Male)^3^, and demand characteristics (0 = not understood; 1 = understood) as predictors and the size of the AC effect for each participant as the outcome (i.e., the difference between health ratings for Stimulus A, which was always paired with the healthy US, and Stimulus B, which was always paired with the unhealthy US).

The regression model was not significant, *F*(8, 444) = 1.19, *p* = .30, *R*^2^ = .02, 95% CI = [−.01, .05], adjusted *R*^2^ = .00, BF_10_ = .00, and each of the predictors were also nonsignificant: openness (β = .00, *p* = .91, BF_10_ = .11), conscientiousness (β = −.03, *p* = .55, BF_10_ = .11), extraversion (β = −.02, *p* = .67, BF_10_ = .10), agreeableness (β = .07, *p* = .17, BF_10_ = .31), neuroticism (β = −.04, *p* = .51, BF_10_ = .10), age (β = −.05, *p* = .30, BF_10_ = .32), and demand characteristics (β = .00, *p* = .91, BF_10_ = .11), with the exception of sex (β = −.11, *p* = .03, BF_10_ = .1.48) where females showed stronger AC than males, although this was a weak effect.

## Discussion

None of the Big Five personality traits predicted the AC effect, which contrasts with two recent studies that examined the relationship between the Big Five personality traits and EC effects (Ingendahl & Vogel, 2023; Vogel et al., 2019). Participants’ age and awareness of the aim of the experiments (i.e., demand characteristics) also did not predict the AC effect. However, females showed a stronger AC effect than males, although this was a weak effect.

## General Discussion

The current experiments examined whether the AC effect is sensitive to cue competition effects (i.e., blocking and overshadowing), the Big Five personality traits, and other individual differences (i.e., age, sex and demand characteristics). An AC effect was observed with the attribute of health being conditioned to neutral stimuli. The AC effect also impacted on participants’ ratings of CSs for nonconditioned attributes (i.e., athleticism, education, humor, and organization). However, there was no evidence of cue competition effects or personality traits impacting the AC effect. Participants’ age and awareness of the purpose of the AC task also did not seem to influence the AC effect. However, females did show a greater AC than males, although this was a weak effect. To our knowledge, these findings are the first to examine cue competition effects with AC using large samples (and control trials) and the first to explore the relationship between personality traits and other individual differences and the AC effect.

The AC effect observed in this study is consistent with studies that have demonstrated AC effects across a range of experimental paradigms and with a variety of different attributes (Unkelbach & Högden, 2019). The observation that CSs paired with healthy foods were also viewed as being more athletic, educated, and organized—but less humorous—than CSs paired with unhealthy foods suggests there is also an indirect conditioning effect of AC. Given that ratings for humor were higher for the unhealthy CS than the healthy CS, it appears this indirect effect is not underpinned by a halo effect (Nisbett & Wilson, 1977). Instead, our findings suggest that stereotypes may be formed which bias participants’ ratings toward other nonconditioned attributes in specific directions based on the attribute that has been conditioned. For example, our results are consistent with previous studies into consumption stereotypes. Barker et al. (1999) reported that individuals who consumed healthy low-fat diets were perceived as being intelligent and serious, whereas those with unhealthy diets were perceived to be less intelligent but more sociable and fun.

The absence of cue competition effects in our study is consistent with similar studies into EC (Dwyer et al., 2007) and AC (Förderer & Unkelbach, 2015). In both our experiments, there was little evidence of blocking and overshadowing, in fact there was slight evidence of augmentation in Experiment 1 (which has also been observed in EC experiments; Beckers et al., 2009). The absence of cue competition effects brings into question the validity of the signal learning account. According to this account of AC, cue competition effects would be expected as the statistical contingency between the blocked and overshadowed cues should be diminished relative to the respective control stimuli. Our results are, however, consistent with a referential account (Baeyens et al., 1992) which has been applied to account for AC (and EC) effects (Unkelbach & Förderer, 2018). According to this account, AC effects are underpinned by a *referential system* where the CS simply prompts a representation of the US (i.e., the attribute) without prompting an assumption that the US will then be presented. The referential system is not sensitive to the statistical contingency between stimuli but simply to the co-occurrences of stimuli (Baeyens et al., 1992). This explanation can therefore account for the absence of cue competition effects in AC and EC tasks as all CSs co-occurred with the USs although the contingencies vary.

Similarly, propositional or reasoning-based explanations could also account for our results (e.g., De Houwer, 2009; Mitchell et al., 2009). According to the propositional account, participants form propositional statements, or beliefs, about the relationships between CSs and USs. For example, participants could infer that CS A *co-occurs* with US1 or that CS A *causes* US1. The nature of these propositional statements (e.g., whether a CS co-occurs or predicts a US) influences how participants understand the relationship between stimuli and may determine whether cue interaction effects such as blocking and overshadowing are likely to occur. For instance, if CS A is deemed to *predict* US1, this propositional belief may result in CS A “blocking” an association from being formed between an additional CS and the same US, as learning about the additional CS is considered unnecessary. If, however, CS A is simply deemed to co-occur with US 1, this could enable participants to learn equally well (or indeed support learning, e.g., Walther et al., 2011) about the relationship between an additional CS and the same US. That is to say, if CS A and US1 are simply deemed to co-occur, this leaves room for an additional CS to enter into a similar, or possibly even stronger, relationship with US 1.

The relationship between personality traits and AC has not been previously explored to our knowledge. However, two recent studies (Ingendahl & Vogel, 2023; Vogel et al., 2019) have identified that neuroticism and agreeableness were associated with EC. Given these findings, it might be surprising that a similar set of results were not identified in relation to AC. It is worth noting, however, that although AC and EC are conceptually similar, they are deemed to be orthogonal effects (Unkelbach & Högden, 2019) and thus may share different relationships with the Big Five personality traits. Recall that in EC the *valence* of a neutral stimulus is changed (De Houwer et al., 2001). However, in AC, the neutral stimulus is imbued with a specific attribute (e.g., healthiness), yet the valence of the stimulus may remain unchanged (see Förderer & Unkelbach, 2014). It is thus plausible that the nonevaluative nature of AC, in which the valence of stimuli remains unchanged, prevents agreeableness and neuroticism from influencing the AC effect in the same manner as they do the EC effect (Casini et al., 2023; Ingendahl & Vogel, 2023). Future studies could further explore the relationship between AC and personality at the *facet* level of the Big Five traits, as has been done with EC (i.e., Ingendahl & Vogel, 2023). Participants’ awareness of the purpose of the AC task also seemed to have no impact on whether an AC effect was observed. Participants’ age also did not influence the AC effect; however, the age range in the study was limited. Females did show stronger AC effects than males although this was a weak effect. Interestingly, EC effects have not been shown to be influenced by participants’ sex (Hofmann et al., 2010), perhaps indicating another way in which these two effects differ from one another.

Taken together, the results of the current study show that attributes can be conditioned, using a remote online task, and this effect is insensitive to a variety of factors that typically influence other forms of conditioning. These findings have implications for marketing techniques and strategies that are commonly used by businesses and organizations. Businesses commonly use celebrities and brand ambassadors to endorse and promote their products or services in the hope that the attributes of these figures are conferred to their products (Knoll & Matthes, 2017). The current studies demonstrate this could be a powerful strategy if the right individuals are identified (e.g., the individual is synonymous with a specific attribute the company wish to invoke). In our studies, simple co-occurrences were sufficient for neutral CSs to be imbued with the conditioned attribute and even nonconditioned attributes that might be indirectly associated with the conditioned attribute. Furthermore, that blocking and overshadowing were not apparent in AC is useful in this context as a new affiliate company, for example, can join an existing brand that is associated with an attribute or value and co-market with it, in the absence of fear that the two companies or brands would compete for the attribute. Future research could examine this in more authentic advertising and marketing contexts. Interestingly, Eckmann et al. (2024) have recently examined AC in marketing contexts. They found that pairing a single attribute with a brand is more effective at establishing an attribute–brand association than pairing multiple attributes with a brand. Theoretically similar studies on multiple outcome learning have also been reported (Quigley & Haselgrove, 2020, 2023).

There are some limitations to the current study though. First, during the preratings stage where participants were asked to rate the healthiness of stimuli, we may have inadvertently primed participants to think about healthiness inflating the possibility of demand characteristics influencing the effect. However, we did assess the possible impact of demand characteristics in our experiments and found that they did not influence the presence of an AC effect. We also did not include a semantic primary task which has been included in some previous studies (Förderer & Unkelbach, 2011) and our sample also contained self-report measures. Furthermore, our sample also largely consisted of university students with a relatively restricted age range which therefore limits the generalizability of our results.

In conclusion, the current study identified an AC effect with the attribute of health. The AC effect also impacted on participants’ ratings of CSs for nonconditioned attributes. However, the AC was insensitive to cue competition effects and personality traits, but there was an effect of sex. These results demonstrate that the AC effect is a robust phenomenon that is insensitive to factors that typically influence other forms of conditioning.

## Notes

1. A directional BF which assumes the presence of blocking revealed BF+_10_ = .03.

2. A directional BF which assumes the presence of blocking revealed BF−_10_ = .02.

3. Five participants identified as nonbinary and were not dummy-coded in the regression model.
